# Discomfort improvement for critically ill patients using electronic relaxation devices: results of the cross-over randomized controlled trial E-CHOISIR (Electronic-CHOIce of a System for Intensive care Relaxation)

**DOI:** 10.1186/s13054-022-04136-4

**Published:** 2022-09-03

**Authors:** Lili Merliot-Gailhoustet, Chloé Raimbert, Océane Garnier, Julie Carr, Audrey De Jong, Nicolas Molinari, Samir Jaber, Gerald Chanques

**Affiliations:** 1 Department of Anaesthesia & Critical Care Medicine, Saint Eloi Montpellier University Hospital, and PhyMedExp, University of Montpellier, INSERM, CNRS, Montpellier, France; 2 Department of Statistics, CNRS, Institut Montpelliérain Alexander Grothendieck (IMAG), University of Montpellier La Colombière Hospital, University of Montpellier, Montpellier, France

**Keywords:** Virtual reality, Music therapy, Non-pharmacological therapies, Supportive care, Intensive care

## Abstract

**Purpose:**

To assess the impact of different electronic relaxation devices on common stressful patient symptoms experienced in intensive care unit (ICU).

**Methods:**

Sixty critically ill patients were enrolled in four relaxation sessions using a randomized cross-over design: standard relaxation (TV/radio), music therapy (MUSIC-CARE©), and two virtual reality systems using either real motion pictures (DEEPSEN©) or synthetic motion pictures (HEALTHY-MIND©). The goal was to determine which device was the best to reduce overall patient discomfort intensity (0–10 Numeric Rating Scale (NRS); primary endpoint). Secondary endpoints were specific stressful symptoms (pain, anxiety, dyspnea, thirst, and lack of rest feeling) and stress response measured by Analgesia/Nociception Index (ANI). Multivariate mixed-effect analysis was used, taking into account patient characteristics and multiple measurements.

**Results:**

Fifty patients followed the full research protocol, and ten patients did at least one research planned session of relaxation. HEALTHY-MIND© was associated with a significant decrease in overall discomfort, the primary endpoint (median NRS = 4[2–6] vs. 2[0–5]; *p* = 0.01, mixed-effect model), accompanied by a significant decrease in stress response (increase in ANI, secondary endpoint; *p* < 0.01). Regarding other secondary endpoints, each of the two virtual reality systems was associated with a decrease in anxiety (*p* < 0.01), while HEALTHY-MIND© was associated also with a decrease in pain (*p* = 0.001) and DEEPSEN© with a decrease in lack of rest (*p* = 0.01). Three incidents (claustrophobia/dyspnea/agitation) were reported among 109 virtual reality sessions. Cybersickness was rare (NRS = 0[0–0]).

**Conclusion:**

Electronic relaxation therapy is a promising, safe, and effective non-pharmacological solution that can be used to improve overall discomfort in alert and non-delirious ICU patients. Its effectiveness depends on technical characteristics (virtual reality using a synthetic imagined world versus a real world or music therapy alone without virtual reality), as well as the type of symptoms.

## Key messages

- Electronic relaxation therapies are effective supportive care tools for improving stressful symptoms in ICU patients.
- Effectiveness depends on the type of symptom and the characteristics of the devices.
- Overall discomfort and adrenergic stress response are more significantly improved by virtual reality using a synthetic imagined world than using a real world or music therapy alone.

## Introduction

Admission in an intensive care unit (ICU) is associated with many causes of suffering, related to the illness or intensive therapies and environment [1, 2]. The consequences can be serious, from the development of an adrenergic stress response interfering with critical illness (tachycardia, polypnea, patient/ventilator asynchrony, agitation, immunosuppression, etc.) [3, 4] to the development of a post-intensive care syndrome (PICS), including neuro-psychological disorders (anxiety, depression, post-traumatic stress syndrome) and chronic pain that delay the return to a social and professional normal life [5, 6]. The link between suffering in ICU and the development of PICS has been highlighted for a long time [7], leading to conceptualize modern intensive care as the most humane possible [8, 9]. For all these reasons, supportive care has become part of intensive care, along with the treatment of organ dysfunctions. In order to prevent drug-related side effects that can be serious in critically ill patients [10], current guidelines for the management of pain, agitation, delirium, immobility, and sleep disruption (PADIS) suggest to develop non-pharmacological therapies [1, 11].

Electronic innovative technology has been developed in medical settings, such as music therapy using programmable shape and length of music score [12, 13], and immersive virtual reality (VR), to offer a non-pharmacological treatment of pain and anxiety [14]. The literature regarding the use of VR in medical care is beginning to be abundant, but its use to relieve overall discomfort and baseline stressful symptoms in ICU has never been the object of a randomized study.

In this context, we propose to assess the impact of different electronic relaxation devices to improve ICU patients’ stressful symptoms, through a cross-over randomized study. The primary goal was to determine the best technique to improve overall patient discomfort (primary endpoint), among 3 different relaxation devices (VR system with real motion pictures, VR system with computer-generated pictures, music therapy provided through a computer and an headset), compared to a standard relaxation session (TV, radio available at bedside). Secondary goals were to measure the impact on 5 of the main stressful symptoms experienced by ICU patients (pain, anxiety, dyspnea, thirst, and lack of rest) and on electrophysiological measurement of stress response using the Analgesia/Nociception Index (ANI) and usual physiological variables (secondary endpoints).

## Material and methods

### Ethics approval

This randomized controlled trial (RCT) was approved by an ethics committee [*Comité de Protection des Personnes Sud-Méditerranée-1* (ID-RCB:2016-A00748-43)] according to French law [15] and registered on Clinical Trials NCT04017299, 12 July 2019. The research was funded by the French healthcare ministry (Direction Générale de l’Offre de Soins (DGOS)/General Healthcare Supply Direction).

### Patient population

The trial took place in the medical-surgical ICU of the University Hospital of Saint Eloi, Montpellier, France, from July 2019 to December 2019.

All consecutive patients over 18 yo admitted to the ICU were eligible if they were alert and non-delirious, following the French validated versions of the Confusion Assessment Method for the ICU (CAM-ICU) [16, 17] and the Richmond Agitation Sedation Scale (RASS) that had to be ≥ 0 [18], and with current organ dysfunctions defined by a Sequential Organ Failure Assessment (SOFA) score ≥ 3 [19].

The exclusion criteria were: patient transferred from another ICU, dying patients, patients needing hygiene precautions limiting the access to the headsets, patients usually treated by antipsychotics or with previous known cognitive impairment, patients hospitalized for brain injury, and according to French law [15]: pregnant or breastfeeding women, patients under tutelage or curatorship, patients not registered with the national social security system, or who refused to participate.

### Conduct of the study and description of the electronic relaxation tools

Patients were screened daily for eligibility by investigators and included in the study after written consent. Patients underwent four relaxation sessions consecutively, after randomization of the session order, using a cross-over design. The cross-over randomized design was chosen to take into account the possible impact of the session order on the outcomes, the subjective nature of the measurement, and the possible cumulative effect on memory for repeated sessions during a short time (intensive care). The randomization was made by the statistic department. The randomization table was generated by randomly drawing the permutations between the 4 interventions (relaxation sessions). The order of the 4 relaxation sessions was provided via sealed numbered envelopes available for the ICU investigators who opened the envelopes after enrolling the patient consecutively. At least one hour of washout was planned between two sessions. The four relaxation sessions are described as follows:

- *STANDARD SESSION*: a relaxation and distraction period of 15 min during which usual distraction was chosen by the patient (e.g., television, radio). The bedroom’s door was kept closed, and any nursing or medical procedures of care were prevented during the session.
- *MUSIC-CARE© *(Paris, France)**:** a 15-min relaxation session of psycho-musical intervention (music therapy) using the MUSIC-CARE© device. As described previously [12], this device provides pieces of music played by musicians, with music score’s components (tempo, intensity, number of instruments) decreasing slowly until a minimal stage, and re-increasing just before the end of the relaxation session (U-shaped score). The duration of the session is modifiable. Music therapy was provided through a high-quality audio headset, the style of music being chosen by the patient from the range offered by the software available at each bed-side computer, with the duration of the session set at 15 min.
- *HEALTHY-MIND© VR system* (Paris, France)**:** a 15-min relaxation session of VR immersing the patient into an artificial world created from computer-generated pictures using a software linked to the Occulus Rift© helmet and a headset. The patient is able to choose four kinds of environments (beach, mountain, snow and Japan) using synthetic motion pictures, breathing exercises and hypnotic speech, as proposed by the manufacturer.
- *DEEPSEN© VR system* (Saint-Didier-au-Mont-d’Or, France)**:** a 15-min relaxation session of VR, immersing the patient into a series of filmed sequences, using an “all-in-one” headset created by the company. The patient was able to choose the film among four kinds of videos (Norway, mountain countryside, India, and Camargue, a quiet seaside in South of France, near Montpellier) using real motion pictures, breathing exercises, and hypnotic speech, as proposed by the manufacturer.

**Fig. 1:**
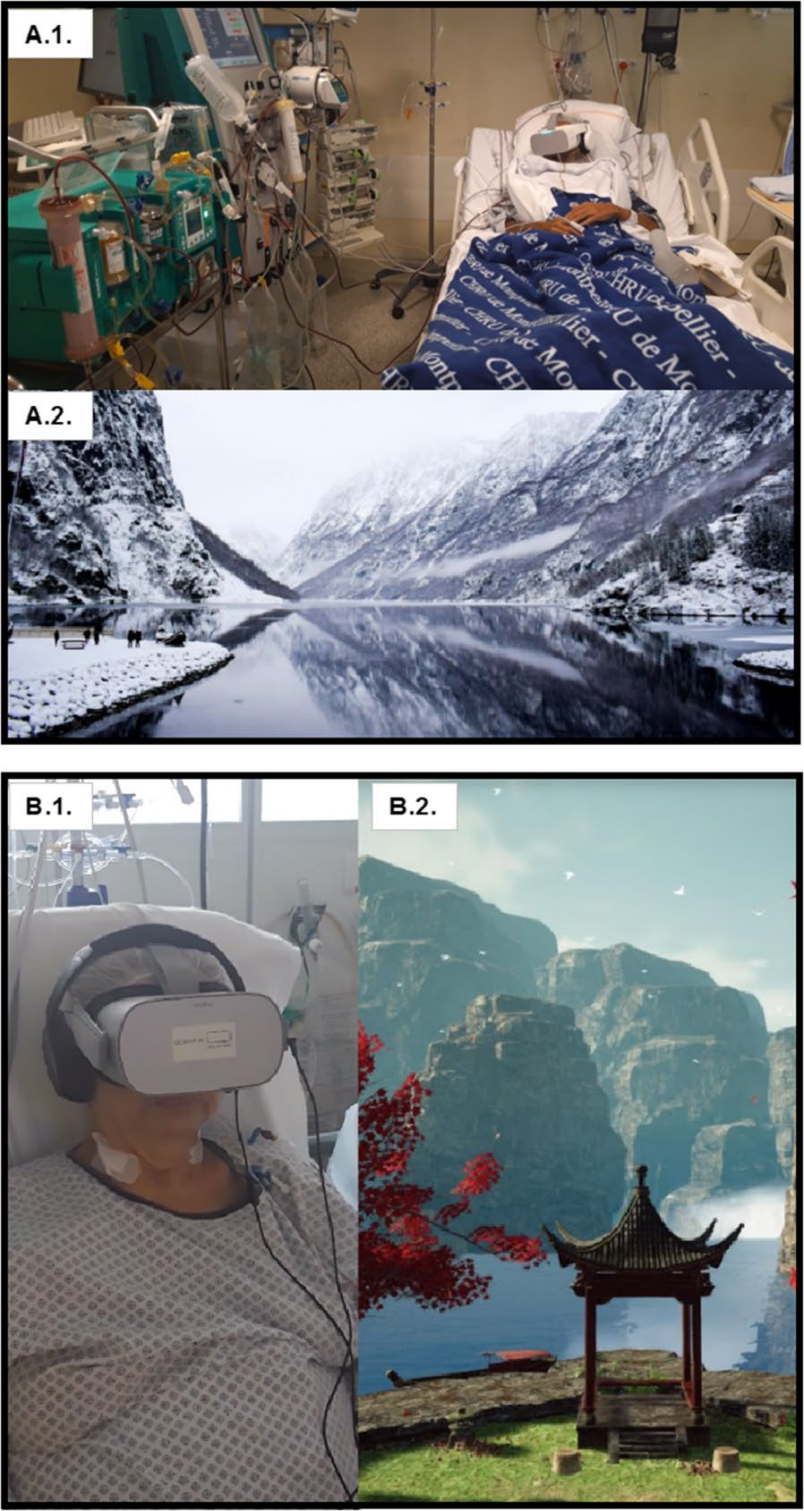
Examples of virtual reality’s worlds. A.1. ICU patient with DEEPSEN © Virtual Reality headset. A.2. DEEPSEN© provides motion pictures from real environment, here Norway. B.1. ICU patient with HEALTHY-MIND© Virtual Reality headset. B.2. HEALTHY-MIND© provides synthetic motion pictures from imagined environment, here Japan

Figure 1 shows an example of virtual reality’s world and headsets. Headsets and headphones were cleaned before and after each session. Protections were placed between the device and the patient.

### Data handling

After enrollment in the study, a relaxation technique was proposed to the patient at the time of the day she or he preferred, from morning to afternoon, in the order of randomization. At baseline, before starting the session, the following vital variables were recorded from the patient’s bedside monitoring: heart rate, blood pressure, respiratory rate, and Analgesia/Nociception Index (ANI). ANI measures heart rate variability from the bed-side electrocardiographic monitoring of the patient, providing a number from 0 to 100 through spectral analysis. This number is an estimation of the balance between parasympathetic and sympathetic outflows: 100 means a high parasympathetic modulation (low stress level = low risk of suffering) and 0 means extremely low parasympathetic modulation (high stress level = high risk of suffering) [20]. Only instant-ANI (PhysioDoloris© monitor, MDoloris Medical Systems, Lille, France) was recorded because it provided an analysis of heart rate variability on 64 s compared to 4 min for mean-ANI. It had previously been reported that instant-ANI was more accurate than mean-ANI in ICU patients [20, 21]. Delirium was assessed before each session by the CAM-ICU. In the absence of delirium, the relaxation session was provided and the patient was asked to report her or his symptoms’ intensity before and after the session using a large visual Numerical Rating Scale (NRS) ranging from 0 to 10 (0 = no symptom at all; 10 = the worst possible symptom). This scale is the most valid self-report pain scale in ICU patients [22] and is therefore the tool recommended to assess pain intensity in ICU patients who are able to communicate [1, 23]. Intensities of other stressful symptoms can also be measured using self-report pain scales such as the 0–10 NRS [2] and were currently used in studies investigating thirst [24], anxiety [25, 26], dyspnea [25], and sleep privation [1]. Overall discomfort (physical and psychological discomfort) and five current stressful symptoms were assessed: pain, anxiety, thirst, dyspnea, and lack of rest. All parameters were recorded again after the end of each relaxation session.

### Statistical analysis

#### Primary endpoint

Variation in the intensity of overall patient discomfort before and after each relaxation session to determine which technique was the most effective to reduce discomfort.

#### Exploratory secondary endpoints

##### Secondary endpoint related to patient’s suffering

Variation in the intensity of five stressful symptoms: pain, anxiety, dyspnea, thirst, lack of rest [2]

##### Secondary endpoints: physiological stress response

Variation in physiological variables (heart rate, arterial blood pressure, respiratory rate), and ANI, as an electrophysiological parameter related to the stress response.

#### Safety and feasibility

The intensity of cybersickness (defined as a visuospatial vertigo associated or not with nausea and vomiting) was self-reported by the patient after the relaxation session. The feasibility of the relaxation technique was assessed by the investigation team using the 0–10 NRS to report the ease of device installation/setting for the investigators (from 0 “not easy at all” to 10 “the easiest possible”). The investigation team collected also any incidents that occurred during the relaxation sessions.

#### Number of patients needed to be enrolled

Taking into account the exploratory nature of the study, the sample size was calculated based on the number of possible orders of relaxation sessions that was calculated as follows: 4 types of sessions × 3 × 2 = 24 possible orders of sessions. Two different patients were expected to be enrolled at least for each kind of sequence (i.e., 48 patients), to allow for a homogenous repartition of different possible relaxation orders among the studied population. To take into account lost to follow-up patients and missing data, 60 patients were expected to be enrolled in the study.

#### Presentation of data and analysis

Description of the data was made using usual descriptive statistics for quantitative variable (mean (standard deviation) or median [25th–75th percentiles] depending on the distribution of the data) and for qualitative data (*n*, number of data available (frequency in percentage)). All relaxation sessions were included in intent-to-treat analysis. Univariate analysis was carried out to compared data «before» versus «after» each relaxation sessions for NRS, ANI, and physiological variables, using the Student’s *t* test or the Wilcoxon–Mann–Whitney’s *U* test according to the distribution of data.

The comparison of electronic techniques between them and the standard relaxation session was carried out by a mixed-effect multivariate analysis, adjusted on session’s order and significant patient’s characteristics, to take into account potential biases (fixed factors) and repeated measurements (patient was considered as the random factor). A backward selection was made manually by removing at each step the variable with the highest *p*-value to ultimately keep only the significant variables (*p* < 0.05) in the model. The type of relaxation technique and the session order were always maintained in the model during the selection.

For all statistical tests, a *p* value of 0.05 was considered statistically significant. Data were analyzed by an independent statistician using the SAS Enterprise Guide version 9.4 (SAS Institute, Cary, NC).

## Results

### Patient baseline characteristics

**Fig. 2:**
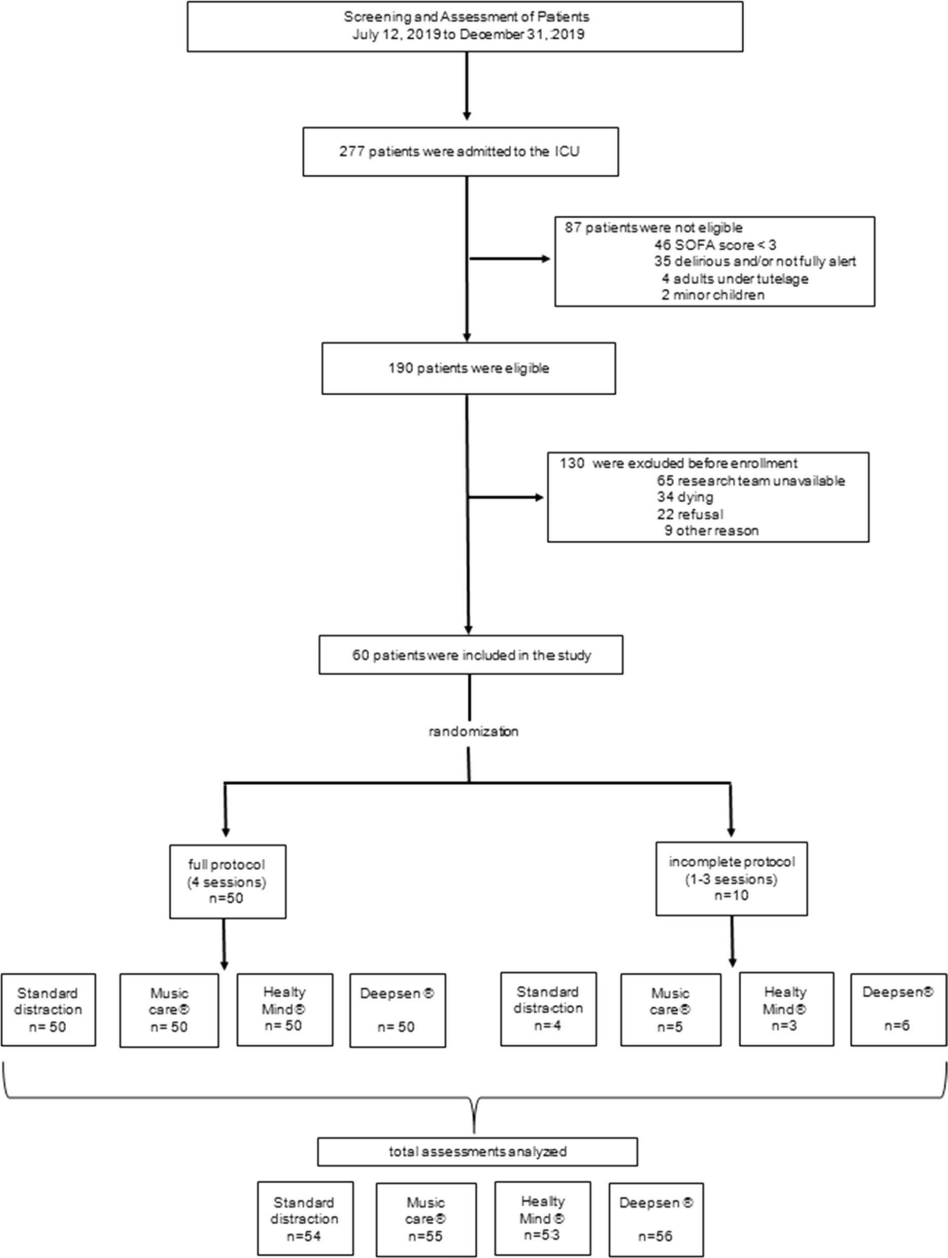
Study flow chart

Among the 277 patients admitted to the ICU during the study period, 190 were eligible and 60 were enrolled**. **Figure 2 shows the study flow chart. Fifty patients (83%) achieved the full protocol (four relaxation sessions), and ten patients performed one to three sessions. Reasons to not perform all sessions were mainly sight problems, claustrophobia, patients who became too tired or delirious (CAM-ICU +), or who left the ICU before the end of the protocol. Patient demographic and medical characteristics are summarized in Table 1. Briefly, patients were admitted to the ICU for a surgical reason in 58% and for a medical reason in 42%. Multimodal analgesia was used preferentially with a minimal use of major opioids and a short duration of sedation in order to optimize the liberation from invasive mechanical ventilation. During the first session, 18 patients were assisted with high-flow oxygen therapy, two with non-invasive ventilation (facemask), and one was intubated; two patients were on continuous extra-renal replacement therapy; and seven required vasopressors.

**Table 1:** Demographic and medical characteristics

Medical characteristics upon admission to ICU	
Age (yr)	62 [51–69]
Sex (F/M)	20/40
BMI (kg.m^−2^)	27 [22–30]
SAPS II score	36 [28–44]
SOFA score	6 [4–8]
*Reason for admission to the ICU*	
Liver transplant, *n* (%)	18 (30%)
Post-operative, *n* (%)	17 (28%)
ARDS, *n* (%)	6 (10%)
Hemorrhagic shock, *n* (%)	5 (8%)
Septic shock, *n* (%)	4 (7%)
Others, *n* (%)	10 (17%)
*Characteristics upon enrolment into the study*	
RASS level	0 [0–0]
CAM-ICU negative, *n* (%)	60 (100%)
Length of stay in ICU before enrollment (days)	2.7 [1.1–3.6]
Invasive mechanical ventilation before enrollment, n (%)	36 (60%)
Time between extubation and enrollment (hours)	25 [14–42]
Sedation before enrollment, *n* (%)	36 (60%)
Number of tubes or catheters at inclusion	4 [2–5]
*Medical characteristics of patients on the first relaxation session*	
Mechanical ventilation, *n* (%)	1 (2%)
Non-invasive ventilation, *n* (%)	2 (3%)
High flow nasal oxygen therapy, *n* (%)	18 (30%)
Dialysis, *n* (%)	2 (3%)
Vasopressors, *n* (%)	7 (12%)
Sedation at enrollment, *n* (%)	0 (0%)
Analgesic administration at enrollment, *n* (%)	45 (75%)
Acetaminophen, *n* (%)	19 (32%)
Nefopam, *n* (%)	38 (63%)
Tramadol, *n* (%)	32 (53%)
Major opioid, *n* (%)	2 (3%)
Epidural analgesia, *n* (%)	3 (5%)
Anxiolytic administration at enrollment, *n* (%)	16 (27%)
*Patients’ outcomes*	
Length of stay in ICU (days)	8.0 [5.2–12.1]
Mortality in ICU, *n* (%)	3 (5%)
Total length of stay in hospital (days)	21.8 [12.6–39.3]
Total mortality in hospital, *n* (%)	7 (12%)

Continuous data are expressed in median [25th–75th percentiles].

*BMI* Body mass index; *SAPS II* Simplified acute physiology score II; *SOFA* Sequential organ failure assessment score; *RASS* Richmond agitation-sedation scale; *CAM-ICU* Confusion assessment method for the ICU; *ARDS* Acute respiratory distress syndrome

### Impact of relaxation sessions on overall discomfort (primary endpoint)

In univariate analysis, HEALTHY-MIND© VR system was associated with significant decrease in overall discomfort (median NRS = 4 [2–6]) before the session versus 2 [0–5] after the session, *p* = 0.02)*.* The two other specific relaxation techniques and the standard relaxation were not associated with significant changes (Table 2). These results were confirmed by mixed-effect multivariate analysis, showing that HEALTHY MIND© VR system was the only relaxation technique associated with a significant decrease in overall discomfort (reduction by 0.8 point; *p* = 0.01, mixed-effect model) (Table 3).

**Table 2:** Symptoms, Analgesia Nociception Index, and physiological variables recorded before and after each of the four relaxation sessions

	Type of relaxation techniques							
	Standard		Music care^®^		VR Deepsen^®^		VR Healthy mind^®^	
*Symptoms*								
NRS overall discomfort								
Before	4	[2;6]	4	[2;6]	5	[2;6]	4	[2;6]
After	4	[2;5]	2.5	[1;5]	4	[1.5;5]	2	[0;5]
* p*	*0.27*		* 0.06*		*0.21*		***0.02***	
NRS pain								
Before	2	[0;5]	2	[0;5]	3	[0;5]	2.5	[0;5]
After	2	[0;5]	1	[0;5]	2	[0;4]	1	[0;4]
*p*	*0.7*		*0.45*		*0.3*		*0.12*	
NRS anxiety								
Before	2.5	[0;5]	3	[0;5]	3	[0;5]	3	[0;5]
After	2	[0;4]	2	[0;5]	1	[0;4]	1.7	[0;4]
*p*	*0.52*		*0.32 *		***0.03***		***0.05***	
NRS thirst								
Before	6	[1;9]	6	[1;8]	5	[1;8]	4	[0;7]
After	6	[0;8]	5	[1;8]	4	[0;8]	4	[0;6]
* p*	*0.68*		*0.53*		*0.38*		*0.93*	
NRS Dyspnea								
Before	1.5	[0;5]	2	[0;5.5]	2	[0;5]	2	[0;4]
After	1	[0;4]	1	[0;4]	2	[0;5]	0.5	[0;4]
* p*	*0.89*		*0.13*		*0.44*		*0.5*	
NRS lack of rest								
Before	4	[2;6]	5	[2;7]	5	[3;8]	5	[3;8]
After	4	[1;6]	4	[0;5]	2	[1;5]	3.5	[2;5]
*p*	*0.49*		***0.05 ***		*** < 0.01***		***0.02***	
*ANI and physiological variables*								
ANI								
Before	62	[48;85]	66	[56;82]	72	[53;80]	67	[51;80]
After	69	[51;82]	82	[65;93]	80	[70;98]	91	[70;98]
*p*	*0.45*		*** < 0.01***		***< 0.01***		***< 0.01 ***	
Heart rate (/min)								
Before	89	[81;102]	88	[80;102]	87	[78;99]	88	[77;100]
After	92	[79;102]	85	[81;101]	84	[74;98]	92	[80;97]
*p*	*0.88*		*0.68*		*0.51*		*0.69*	
Respiratory rate (/min)								
Before	21	[18;24]	20	[17;26]	20	[17;25]	21	[16;27]
After	22	[18;27]	19	[15;24]	21	[17;26]	20	[16;24]
*p*	*0.74*		*0.34*		*0.81*		*0.14*	
Systolic blood pressure (mmHg)								
Before	134	[120;144]	128	[117;154]	136	[121;151]	134	[118;146]
After	133	[119;146]	127	[113;143]	137	[120;151]	131	[115;148]
*p*	*0.99*		*0.51*		*0.86*		*0.82*	
Diastolic blood pressure (mmHg)								
Before	72	[65;82]	71	[62;82]	70	[62;81]	73	[65;82]
After	72	[64;79]	66	[61;77]	75	[61;84]	71	[62;81]
*p*	*0.52*		*0.18*		*0.38*		*0.65*	

The data correspond to a rating from 0 to 10 on a numerical rating scale, where 0 is the best and 10 the worst intensity, and are expressed in median [25th–75th percentiles]. The *p* value was calculated via Mann–Whitney-Wilcoxon test for nonparametric data and via Student’s *t* test for parametric data

The Italic, bold values are for significant *p* values

**Table 3:** Multivariate mixed-effect model—benefits of specific electronic relaxation techniques compared to standard relaxation

	Gain estimate	*p*		Gain estimate	*p*
*Overall discomfort*			*ANI*		
Music care^®^	− 0.4	*0.25*	Music care^®^	0.7	*0.87*
VR Deepsen^®^	− 0.1	*0.85*	VR Deepsen^®^	8.5	*0,07*
VR Healthy mind^®^	− 0.8	***0.01***	VR Healthy mind^®^	13	***0.006***
Standard	0		Standard	0	
*Pain*			*Heart rate*		
Music care^®^	− 0.3	*0.23*	Music care^®^	0.1	*0.39*
VR Deepsen^®^	− 0.3	*0.19*	VR Deepsen^®^	− 0.5	*0.75*
VR Healthy mind^®^	− 0.8	***0.001***	VR Healthy mind^®^	1.0	*0.47*
Standard	0		Standard	0	
*Anxiety*			*Systolic blood pressure*		
Music care^®^	− 0.1	*0.7*	Music care^®^	2.2	*0.7*
VR Deepsen^®^	− 0.9	***0.001***	VR Deepsen^®^	2.1	*0.5*
VR Healthy mind^®^	− 0.8	***0.004***	VR Healthy mind^®^	2.2	*0.8*
Standard	0		Standard		
*Thirst*			*Diastolic blood pressure*		
Music care^®^	0.0	*0.88*	Music care^®^	− 2	*0.2*
VR Deepsen^®^	0.0	*0.93*	VR Deepsen^®^	1.8	*0.2*
VR Healthy Mind^®^	− 0.3	*0.45*	VR Healthy Mind^®^	− 0.3	*0.8*
Standard	0		Standard	0	
*Dyspnea*			*Respiratory rate*		
Music care^®^	− 0.6	*0.06*	Music care^®^	− 1	*0.4*
VR Deepsen^®^	− 0.3	*0.42*	VR Deepsen^®^	0.5	*0.65*
VR Healthy mind^®^	− 0.3	*0.39*	VR Healthy mind^®^	− 2	*0.12*
Standard	0		Standard	0	
*Lack of rest feeling*					
Music care^®^	− 0.7	*0.26*			
VR Deepsen^®^	− 1.6	***0.01***			
VR Healthy mind^®^	− 1.2	*0.07*			
Standard	0				

This multivariable mixed model was performed to compare different electronic devices between them, dealing with multiple assessments, different orders of relaxation sessions, and significant patient characteristics. The estimate of the improvement in the self-reported assessment of symptoms’ intensity, Analgesia/Nociception Index (ANI), and physiological variables was reported for each relaxation device, compared to the standard relaxation session

The Italic, bold values are for significant *p* values

### Impact of relaxation sessions on five common ICU stressful symptoms (exploratory secondary endpoints related to patient’s suffering)

Variation in symptoms’ intensities is shown in Table 2 (univariate analysis) and Table 3 (multivariate analysis). HEALTHY-MIND© VR system was associated with a reduction in pain and anxiety intensities by 0.8 points compared to the standard relaxation (*p* = 0.001 and 0.004, respectively, mixed-effect model). DEEPSEN© VR system was associated with a reduction in anxiety by 0.9 point (*p* = 0.004) *and* a reduction in lack of rest by 1.6 points (*p* = 0.01)*.*

### Impact of relaxation sessions on analgesia/nociception index (ANI) and physiological variables (exploratory secondary endpoints)

ANI increased significantly after each relaxation session except for the standard relaxation (univariate analysis, Table 2). The multivariate analysis showed a significant change for HEALTHY-MIND© VR system only (estimate gain + 13*, p* < 0.01) (Table 3). Other physiological variables did not change significantly.

### Safety

Incidents were reported in three patients during the 109 VR sessions: self-withdrawal related to claustrophobia (*n* = 1) or agitation (*n* = 1) and displacement of the headset to the nose and mouth associated with tachypnea and dyspnea (*n* = 1). In all, premature interruptions of relaxation sessions before the end of the 15-min planned session occurred in 12/218(6%) sessions (3/54(6%), 4/53(8%), 3/56(5%), 2/55(4%), for the standard relaxation, HEALTHY-MIND© and DEEPSEN© VR systems, and MUSIC-CARE©, respectively). Main reasons were eyesight problems, annoyance, interruptions related to visit of patient’s relatives, or urgent care. Cyber-sickness was rarely observed after the use of music therapy or VR headsets, with a median NRS of dizziness of 0 [0–0]. Five on 56 patients (11%) reported a NRS ≥ 4 for dizziness after a session with DEEPSEN© VR system, 2/53 (4%) after a session with HEALTHY-MIND© VR system, 1/55 (2%) after standard session, and none after MUSIC-CARE© relaxation. No other serious side effects were observed. Ten patients (17%) did not complete the four sessions: three because of discharge from ICU, three because of fatigue or anxiety, two because of eyesight problems, one because of the appearance of delirium, and one who developed claustrophobia.

### Feasibility

The placement and use of devices were easy according to the investigators (feasibility-NRS of 10 [10;10] for standard; 10 [9;10] for MUSIC-CARE©; 10 [8;10] for DEEPSEN©; 10 [8;10] for HEALTHY-MIND©). Investigators were present for the placement and configuration of headset. Some patients learned their use very quickly and used it with autonomy after the end of the research protocol.

## Discussion

The main findings of this cross-over randomized study are that using electronic relaxation devices is feasible, safe, and well tolerated in more than 90% of alert and non-delirious critically ill patients. Some devices are more effective than others to relieve ICU patients from stressful symptoms. VR with synthetic motion pictures (imagined world) is the most effective to decrease overall discomfort, compared to real world or music therapy only (primary endpoint). This result is reinforced by a significantly greater increase in ANI with this kind of VR compared to other devices. Exploratory secondary endpoints may explain this result because this relaxation device was also associated with a significant decrease in both pain and anxiety, while the other kind of VR (real world) was associated with a significant decrease in anxiety and lack of rest, but not pain (multivariate analysis). These findings should be discussed and nuanced by the fact that some symptoms (pain, anxiety, dyspnea) had a light intensity (< 4/10) in the included population. These devices could demonstrate different properties in other populations of ICU patients, and these results should not discourage further research in patients with moderate to severe pain, anxiety, or dyspnea. Such research is mandatory because similar relaxation devices may have different impacts depending on the type of symptoms, as reported by the present study.

The study highlights also that today pain is not always the most important stressful symptom in ICU patients, contrary to other symptoms like thirst. Pain management has been improved in ICU for many years [27], including the elaboration of new pain scales [22, 28], the measurement and recognition of pain and related outcomes [29, 30], and the development of practice guidelines promoting multimodal analgesia as the top priority [1]. Pain could be considered as the cornerstone for improvement in patient suffering in ICU. However, other symptoms should be considered [2, 31] and have created more and more interest, like anxiety [26], dyspnea [32, 33], thirst [24, 34], and sleep disruption [1]. It should be noted that for the latter symptom, the intensity of “lack of rest” was measured in the present study instead of “sleep disruption” because the relaxation sessions were organized during the day and not at night.

There has been an increased interest to use non-pharmacological strategies to relieve stressful symptoms in ICU. Listening music was proved to be effective on anxiety [26] while optimizing the ventilator setting was effective on both anxiety and dyspnea [25] and should even be encouraged first before escalating analgesia sedation in mechanically ventilated patients [35, 36]. Sleep and thirst should be facilitated using a bundle of non-pharmacological techniques (e.g., earplugs, eyemask, reduction in noise, light, and care to promote sleep [1], oral swab wipes, ice-cold water sprays, lip moisturizer, mini mint ice cubs to minimize thirst [24, 34]).

The present study reports promising results regarding the use of VR to improve anxiety (both devices), pain (imagined world), or lack of rest (real world). Previous studies reported that music therapy could improve anxiety [26] and pain in ICU [12, 37]. In the present study, MUSIC-CARE© was the only device associated with a possible effect on dyspnea, with a trend toward significance (decrease of 0.6 additional point, p = 0.057, multivariate analysis, Table 3). In a larger population or in patients with higher dyspnea intensity, this technique may demonstrate significant improvement. This is consistent with a previous report by our group that observed significant improvement in ventilatory parameters with MUSIC-CARE© in intubated patients during the weaning of mechanical ventilation [12].

The present study reports that devices using both music and VR could be more effective than music therapy alone on overall discomfort and specific symptoms. Strong scientific arguments supporting the beneficial effect of VR have recently spread in medical sphere, especially for the management of mental health disorders including anxiety [38]. Results are promising to manage pain after surgery, severe burns [39, 40], or before nociceptive procedures [41]. Hypothesis is that VR, by providing multisensory inputs, deflects attention into the virtual world and lowers the pain intensity [42]. Thus, the kind of virtual world may play a role as suggested by our results. However, patient characteristics that were not investigated in this study (preference for real or computed generated pictures for example) should be taken into account as potential bias when interpreting the results.

The present study is the first randomized study about VR evaluating the improvement in overall patient baseline comfort in a mixed medical-surgical ICU, compared to music therapy and standard relaxation. A recent review on VR in ICU reported 21 studies, mostly of them being observational, non-comparative, feasibility studies [43], whose reports are consistent with the present study with a completion rate of 74%, (95%CI 51%-96.0%). In two proof-of-concept studies designed by the same group, heart rate and blood pressure decreased in 37 patients [44], while respiratory rate and discomfort decreased in 33 patients [45]. Physiological variables did not change in the present study. This may be explained by differences in the severity of critical illness among studied populations. Usually, physiological variables may change only a little in critically ill patients compared to behavioral pain scales [46, 47] or heat rate variability devices [20]. Among RCTs, one assessed the type of computer-generated video (urban *versus* mountain world *versus* a video displayed on the bedroom-TV screen) on 45 patients, reporting a better restoration feeling with the mountain world [48], consistently with findings of the present study that highlights a different impact of VR related to device characteristics. Three RCTs included 48(24/group) [49], 100(25/group) [50], and 200(100/group) [51] post-cardiac surgery patients. VR improved significantly sleep [49] and relaxation [50] but failed to improve anxiety and pain at rest [50] or during chest drain removal [51]. In the latter study, VR was compared to nitrous oxide. Another negative RCT investigated the impact of VR on PICS in 89 patients with COVID-19 [52].

The present study presents some strengths and limitations. Strengths include the prospective recording of parameters by a research team who assured non-disturbance of care during the relaxation time, the cross-over randomized design, the multivariate analysis that was performed as a method to compare different electronic devices between them, dealing with multiple assessments, different orders of relaxation sessions, and the characteristics of patients that were diverse by nature in a mixed medical-surgical ICU population, as well as the measurement of the most common stressful symptoms [2] using validated complementary subjective and objective tools [20, 22]. Regarding the study limits, as previously discussed, a lack of power related to only moderate symptoms intensities could explain the lack of significance for some results. This study should be reproduced in patients with clinically significant symptoms (i.e., pain, anxiety, dyspnea), and calibrated on each of these stressful symptoms. However, although the absolute values of symptom improvement were rather small, it is important to note that all the results pointed in the same direction: a specific relaxation device, such as music therapy or VR, can improve self-reported patient symptoms and patient stress response. Finally, these results may be biased by the fact that much attention was paid to enrolled patients during the study: multi-daily visit, dialogue, taking into account diverse symptoms and pain, with multiple assessments by nurses and physicians, followed by frequent multidisciplinary management as basically performed for pain according to the guidelines. Also, only a selected group of ICU patients was included; specifically, they were alert and non-delirious, taken in charge in a ICU where a strategy of fast liberation from invasive mechanical ventilation was implemented [35], including a minimal use of major opioids and sedatives according to current guidelines [1, 8, 36]. Thus, further studies are needed to measure the impact of electronic devices in ICU at a large scale, in multiple centers with diverse ICU populations and cultures, and to explore all different characteristics of the devices and their impact on short and long-term outcomes. Because managing ICU patients who are often weak or tired is challenging, these findings could also promote the evaluation of these innovative devices to help managing diverse stressful symptoms in other hospital settings or at home, where they could be easier to implement that in the ICU. Finally, one important finding highlighted by the exploratory part of the study (specific symptom analysis) is that pain might not be the main stressful symptom in critical illness. Attention to pain management in ICU has been placed as the top priority in guidelines for many years but should not mask other significant stressful symptoms. Such symptoms, diverse by nature, deserve further investigations.

## Conclusion

This first RCT investigating different VR devices compared to standard relaxation and to music therapy shows that VR with computer-generated pictures is the most effective to improve overall discomfort and to reduce the physiological stress response in alert and non-delirious ICU patients. Moreover, the type of virtual world and music therapy may impact the symptoms differently: beneficial effects of these new therapies depend on the device characteristics and the targeted symptoms.

## Data Availability

Data are protected by the Clinical Research Direction of the Montpellier University Hospitals and will be available for research upon request after approval by a French ethics committee according to the law.

## Abbreviations

ANI: Analgesia Nociception Index
CAM-ICU: Confusion assessment method for the ICU
ICU: Intensive care unit
NRS: Numeric Rating Scale
RASS: Richmond Agitation Sedation Scale
SAPS: Simplified acute physiological score
SOFA: Sequential organ failure assessment score
VR: Virtual reality

## Declarations

### Human accordance statement

The study was performed in accordance with the ethical standards as laid down in the 1964 Declaration of Helsinki and its later amendments or comparable ethical standards.
