# Publisher Correction to: Discomfort improvement for critically ill patients using electronic relaxation devices: results of the cross‑over randomized controlled trial E‑CHOISIR (Electronic‑CHOIce of a System for Intensive care Relaxation)

**DOI:** 10.1186/s13054-022-04169-9

**Published:** 2022-09-26

**Authors:** Lili Merliot‑Gailhoustet, Chloé Raimbert, Océane Garnier, Julie Carr, Audrey De Jong, Nicolas Molinari, Samir Jaber, Gerald Chanques

**Affiliations:** 1 Department of Anaesthesia & Critical Care Medicine, Saint Eloi Montpellier University Hospital, Montpellier, France, and PhyMedExp, University of Montpellier, INSERM, CNRS, Montpellier, France; 2 Department of Statistics, CNRS, Institut Montpelliérain Alexander Grothendieck (IMAG), University of Montpellier La Colombière Hospital, University of Montpellier, Montpellier, France

## Publisher Correction to: Critical Care (2022) 26:263 https://doi.org/10.1186/s13054-022-04136-4

Following publication of the original article [1], the authors identified errors in Table 2, affiliation 1 and the e-mail address of the corresponding author. In Table 2 certain values were not correctly indicated in bold and/or Italics and a value (0.45) was missing at Music care^®^ > NRS pain > *p*.

The correct Table 2 is given hereafter.

**Table 2:** Symptoms, Analgesia Nociception Index, and physiological variables recorded before and after each of the four relaxation sessions

	Type of relaxative techniques							
	Standard		Music care^®^		VR Deepsen^®^		VR Healthy mind^®^	
*Symptoms*								
NRS overall discomfort								
Before	4	[2;6]	4	[2;6]	5	[2;6]	4	[2;6]
After	4	[2;5]	2.5	[1;5]	4	[1,5;5]	2	[0;5]
* p*	*0.27*		*0.06*		*0.21*		***0.02***	
NRS pain								
Before	2	[0;5]	2	[0;5]	3	[0;5]	2.5	[0;5]
After	2	[0;5]	1	[0;5]	2	[0;4]	1	[0;4]
*p*	*0.7*		*0.45*		*0.3*		*0.12*	
NRS anxiety								
Before	2,5	[0;5]	3	[0;5]	3	[0;5]	3	[0;5]
After	2	[0;4]	2	[0;5]	1	[0;4]	1.7	[0;4]
*p*	*0.52*		*0.32*		***0.03***		***0.05***	
NRS thirst								
Before	6	[1;9]	6	[1;8]	5	[1;8]	4	[0;7]
After	6	[0;8]	5	[1;8]	4	[0;8]	4	[0;6]
* p*	*0.68*		*0.53*		*0.38*		*0.93*	
NRS Dyspnea								
Before	1,5	[0;5]	2	[0;5,5]	2	[0;5]	2	[0;4]
After	1	[0;4]	1	[0;4]	2	[0;5]	0,5	[0;4]
* p*	*0.89*		*0.13*		*0.44*		*0.5*	
NRS lack of rest								
Before	4	[2;6]	5	[2;7]	5	[3;8]	5	[3;8]
After	4	[1;6]	4	[0;5]	2	[1;5]	3.5	[2;5]
*p*	*0.49*		***0.05***		** < *0.01***		***0.02***	
*ANI and physiological variables*								
ANI								
Before	62	[48;85]	66	[56;82]	72	[53;80]	67	[51;80]
After	69	[51;82]	82	[65;93]	80	[70;98]	91	[70;98]
*p*	*0.45*		** < *0.01***		** < *0.01***		** < *0.01***	
Heart rate (/min)								
Before	89	[81;102]	88	[80;102]	87	[78;99]	88	[77;100]
After	92	[79;102]	85	[81;101]	84	[74;98]	92	[80;97]
*p*	*0.88*		*0.68*		*0.51*		*0.69*	
Respiratory rate (/min)								
Before	21	[18;24]	20	[17;26]	20	[17;25]	21	[16;27]
After	22	[18;27]	19	[15;24]	21	[17;26]	20	[16;24]
*p*	*0.74*		*0.34*		*0.81*		*0.14*	
Systolic blood pressure (mmHg)								
Before	134	[120;144]	128	[117;154]	136	[121;151]	134	[118;146]
After	133	[119;146]	127	[113;143]	137	[120;151]	131	[115;148]
*p*	*0.99*		*0.51*		*0.86*		*0.82*	
Diastolic blood pressure (mmHg)								
Before	72	[65;82]	71	[62;82]	70	[62;81]	73	[65;82]
After	72	[64;79]	66	[61;77]	75	[61;84]	71	[62;81]
*p*	*0.52*		*0.18*		*0.38*		*0.65*	

The data correspond to a rating from 0 to 10 on a numerical rating scale, where 0 is the best and 10 the worst intensity, and are expressed in median [25th–75th percentiles]. The *p* value was calculated via Mann–Whitney-Wilcoxon test for nonparametric data and via Student’s *t* test for parametric data

The Italic, bold values are for significant *p* values

Table 2, affiliation 1 and corresponding author e-mail address has been updated in this Publisher Correction article and the original article [1] has been corrected. The publisher apologises to the authors and readers for the inconvenience caused by these mistakes.
